# Cobra Cytotoxins: Structural Organization and Antibacterial Activity

**Published:** 2014

**Authors:** P. V. Dubovskii, Y. N. Utkin

**Affiliations:** Shemyakin-Ovchinnikov Institute of Bioorganic Chemistry, Russian Academy of Sciences, Miklukho-Maklaya Str., 16/10, Moscow, 117997, Russia

**Keywords:** antibacterial activity, lipopolysaccharide, peptidoglycan, plasma membrane, three-finger cardiotoxins (cytotoxins), cytolytic cationic peptides

## Abstract

Cardiotoxins (cytotoxins, CT) are β-structured proteins isolated from the venom of cobra. They consist of 59–61 amino acid residues, whose antiparallel chains form three ‘fingers’. In contrast to neurotoxins with an overall similar fold, CTs are amphiphilic. The amphiphilicity is caused by positively charged lysine and arginine residues flanking the tips of the loops that consist primarily of hydrophobic amino acids. A similar distribution of amino acid residues is typical for linear (without disulfide bonds) cationic cytolytic peptides from the venoms of other snakes and insects. Many of them are now considered to be lead compounds in combatting bacterial infections and cancer. In the present review, we summarize the data on the antibacterial activity of CTs and compare it to the activity of linear peptides.

## INTRODUCTION

Cytolytic peptides are present in the venom of snakes and insects. They contain extended hydrophobic regions flanked by positively charged lysine and arginine residues as a structural motif [1]. Cytolytic peptides can be either linear [2-7] or contain disulfide bonds [8]. In the latter case, they can only be β-sheets [9-12] or contain both β-sheet and α-helical regions [13, 14]. The interest in cytolytic peptides stems from the fact that some of them display both antibacterial and anti- proliferative activities [15-18]. They are widely used to design peptides with improved therapeutic indices [19-23]. The design process is typically based on the principle of combining various motifs in one peptide (e.g., the cytolytic motif, the motif inducing membrane fusion, and the one promoting cell penetration). However, the systematic use of such peptides is undermined by their susceptibility to proteolysis in the bloodstream [24, 25]. Therefore, we believe that peptides with compact structures stabilized by one or several disulfide bonds would be of great interest.

Three-finger toxins from cobra venom belong to the family of cytotoxins (cardiotoxins, CT) [12, 26-28] and can kill various types of cells by disrupting their plasma membranes. Studies of CT interaction with model lipid membranes have demonstrated that its mechanism depends on the cytotoxin type: either P or S [29, 30]. The P-type includes CTs with a Pro30 residue at the tip of the second loop; the S-type, those with a Ser28 residue (*Table*). Data on CT interactions with model phospholipid membranes suggest that these toxins destabilize the lipid bilayer of anionic phospholipid-containing membranes [30, 31]. Evidently, in a living cell CTs target the plasma membrane (or the membranes of intracellular organelles) that contain such phospholipids. The interaction of CTs with the components of the surface membrane of eukaryotic cells disrupts its barrier properties and/or leads to the penetration of CT into the cell and its subsequent interaction with organelles, resulting in cell death [32-35]. Presumably, this scenario requires anionic glycolipid sulfatide to be present in the membrane [36]. On the other hand, bacterial cell membranes are almost entirely composed of anionic phospholipids [37] and therefore should be substantially more vulnerable to CTs. The aim of this review is to verify this claim.

**Table:** Cardiotoxins: properties and conformational characteristics

Cobra species, Naja	Abbreviation	Alternative names	ID^1^	I/11^2^	S/P^3^	HTL^4^	Net positive charge (neutral pH)	Method	PDB code^5^
N. mossambica	M1	CTX: IIb, VII1 CT-1	P01467	I	P	3.4	8	NMR	2CCX
	M3	CTX VII4 CT-4	P01470	II	S	1.0	10	XRA	1CDT
N. atra	A1	CTX:-1, I CT:-1(CX1)	P60304	II	S	12.5	7	NMR	2CDX
	A2	CTX:-2, II CT:1A, -2(CX2)	P01442	II	S	12.9	8	NMR	1CRF 1CRE
	A3	CTX:-3, III CT-3	P60301	II	P	11.7	9	NMR	2CRT, 2CRS
								NMR	1I02
								XRA	1H0J
								XRA	1XT3
								XRA	2BHI
	A4	CTX:-4, IV CT:-4	P01443	II	S	12.9	9	NMR	1KBT 1KBS
	A4b	CTX:-A4b; -T CT: D-1; -5	P07525	II	S	9.8	9	NMR	1CHV
	A6	CTX:6, N CT:-6, N	P80245	I	P	9.3	8	XRA	1UG4
N. oxiana	CII (CTII)	CT-2	P01441	II	P	16.3	10	NMR	1CB9, 1CCQ
								NMR	1FFJ
	CII (CTI)	CT-1	P01451	II	S	8.9	6	NMR	1RL5
								NMR	1ZAD
N. pallida	Tg	CTX: gamma CT-1	P01468	I	P	3.4	9	NMR	1CXO
								XRA	1TGX

^1^Code of the amino acid sequence in the Swiss-Prot database of protein structures (www.uniprot.org).

^2^Classification into CT Group I and II is based on the presence of either two Pro (Group I) or a single Pro (group II) residues in the loop I sequence.

^3^Classification of CT into S- and P-type is based on the presence of the S28 and P30 residues, respectively, at the end of loop II.

^4^Residues 5-11, 24-37, 46-50 and the Kyte-Doolittle hydrophobicity scale were used for calculations, a higher value corresponds to a higher hydrophobicity of the HTL.

^5^Protein structure database PDB (www.rcsb.org/pdb/home/home.do).

All CT molecules contain such structural and functional motifs as the membrane-binding motif and a ‘belt’ of charged residues surrounding it, as well as clusters of conserved polar residues [12]. We can expect toxin activity to be defined by the efficiency of these motifs at certain stages of the CT penetration into a bacterial cell, as well as by the succession of their involvement in the interactions with the cell. Let us first examine the spatial structure of a CT molecule.

## SPATIAL STRUCTURE OF A CT MOLECULE

**Fig. 1:**
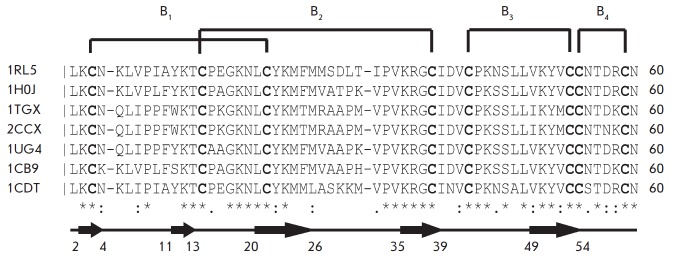
Alignment of the amino acid sequences (cysteine residues are shown in bold) of CTs whose spatial structures have been determined by NMR or X-ray analysis (Table ). Codes of the corresponding structures in the PDB bank are shown on the left. Sequences of asterisks, colons, and dots indicate the following residues: conservative, close and more distant in terms of properties, respectively. Disulfide bonds (B1 to B4) are designated by brackets above the sequence. The secondary structure is given below: sections of antiparallel chains are indicated by arrows under which the boundaries (residue number) of their formation are marked

The researchers working at the Shemyakin-Ovchinnikov Institute of Bioorganic Chemistry of the Russian Academy of Sciences have made a significant contribution to the elucidation of the spatial structure of CT molecules, which has been discussed in several papers [30, 38-40] and reviews [12, 23, 27]. Herein, we present a brief overview. CTs are characterized by a high degree of homology of their amino acid sequences. *Fig. 1* shows the alignment of CT amino acid sequences, whose spatial architecture was determined by X-ray diffraction or NMR. *Table *lists the supplementary data (references, short names, charges of these toxins, etc.).

**Fig. 2:**
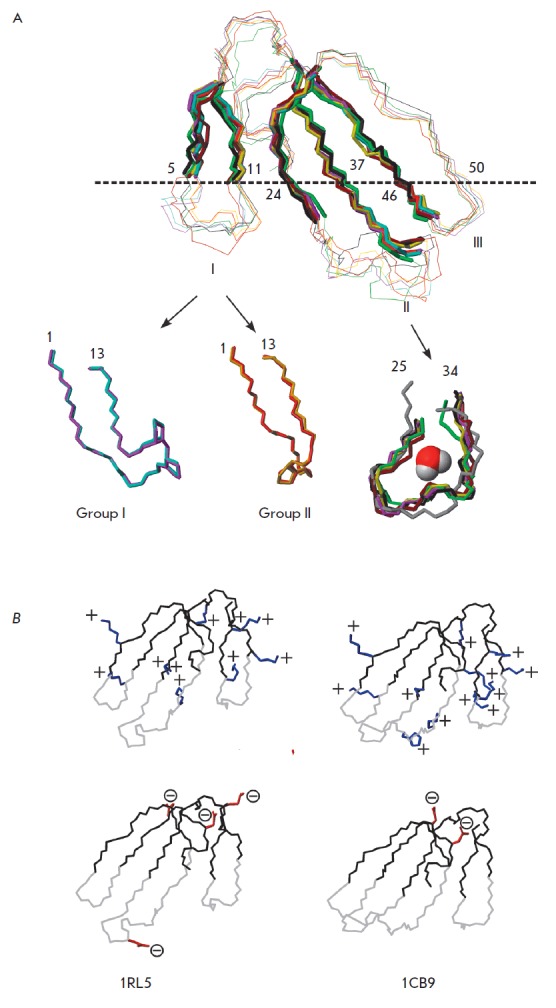
Detailed spatial structure of CTs. A – superposition of structures of cytotoxins from Fig. 1 (details are also given in Table) based on the elements of their secondary structure (strands of antiparallel β-structure are shown in bold). The loops are numbered with Roman numerals. The dotted line shows the boundary between the membrane and water established by NMR in the model system [38]. The numbers of amino acid residues located at this boundary (according to [38]) are given next to the line. Superposition of residues from the first and the second loops is shown at the bottom of the set (only for the crystal structures from the set). For loop I, the superposition of Group I CTs (characterized by the presence of two Pro residues at the tip of the loop) and Group II CTs (single residue at the tip of the loop Pro) is given. Heavy atoms of the side chain Pro residues are displayed. A water molecule is shown in the center of loop II (formation of its hydrogen bonds: see text for details). B – distribution of positive (Lys, Arg, His residues, top, end groups of residues are marked with a “+”, in blue) and negative (Asp, Glu, residues, bottom, end groups of the side chains of these residues are marked with a “-” in a circle, red) charges, exemplified by the structures of CT2 of N. oxiana (left) and CT1 of N. oxiana (right). The set of PDB codes is shown below (representing the structure #1 from the set of 20 deposited structures). Residues of the polypeptide backbone, forming a membrane motif, are shown in gray

All CTs are β-sheet proteins with three-finger folding [41] (*Fig. 2*). Four disulfide bonds formed by eight cysteine residues are the conserved elements of their spatial architecture (*Fig. 1*). It should be noted that the Asn60 residue, located in the immediate vicinity of the last cysteine residue that is conserved in all CTs, plays an important structural role. The side chain of this residue participates in three hydrogen bonds in the hydrophobic core of the molecule.

CT loops are formed by the antiparallel strands of the β-sheets (*Fig. 2*). The sizes of the β-sheets, small (formed by two strands of loop I) and large (formed by both strands of loops II and III), and their twist are sim ilar in different CTs. Dissimilarities in the structures of different CTs are observed in regions with irregular structures, i.e. at the tips of loops I and II. In Group I CTs (this group includes CTs with two Pro residues at the tip of the loop I), this loop is bent (*Fig. 2A*) and has a ‘banana-twist’ shape [42]. It is achieved through two Pro residues at the tip of loop I (the first of which (Pro8) is present in the *cis*-configuration) and type VIa rotation (stabilized by hydrogen bonds between 10 HN ... O = C 7 and side chain NH of the Gln5 residue and C=O of residue 7). In Group II CTs (this group includes CTs with one Pro8 residue at the tip of loop I), this loop is more extended (*Fig. 2A*).

An interesting feature of CTs is the Ω-shape of the loop II tip (*Fig. 2A*). NMR spectroscopy revealed that this region binds a water molecule with a long lifetime of the bound state [43]. This molecule may participate in up to three hydrogen bonds: with one of the amide protons of loop II and with two carbonyl groups of the polypeptide backbone of this portion of the molecule.

The loop III structure is the most conserved one in all CTs. It starts with residues 40–45 (numbers are given for a CT built of 60 amino acid residues) that form a cross-turn with a right twist (*Fig. 2A*). It connects the external polypeptide chains of the three-stranded β-sheet. Residues 46–49 at the tip of loop III form a type I β-turn. Residues 49–54 form an antiparallel strand structure with residues 20–26. It is interesting to note that the length of this final strand is strictly identical in all known structures of CTs [12].

The tips of the loops play the crucial role in the interactions of CTs with detergent micelles and lipid membranes [38, 44]. They form a membrane-binding CT motif (*Fig. 2A*). The degree of hydrophobicity of the residues in this motif can serve as a more subtle basis for CT classification (*Table*, column HTL) [23] than the previously proposed subdivision of CTs into the S- and P-types [29].

In general, the overall positive charge of a CT molecule ranges from 4 to 12 [23]. The difference is due to variations in the ratio between the negatively charged amino acids residues (aspartic and glutamic acids) and the positively charged lysine and arginine residues (*Fig. 2B*). The latter mediate the interactions of CT s with the cell surface polyanionic glycopolymers of animal cells, glycosaminoglycans (GAGs) [45]. Charge distribution in a CT molecule defines the corresponding association constant [46].

The biological effect of CTs on various types of cells is mediated by: 1) the interaction with the components of the cell wall (if any) and the plasma membrane; 2) penetration into a cell; and 3) the subsequent interaction with cellular organelles.

## ANTIBACTERIAL ACTIVITY OF CTS

The venoms of snakes and insects have long been considered to be a source of various biologically active compounds [47-55], including antibacterial ones. The low incidence of infections in snake bite wounds is a clear indication that the venom includes antibacterial compounds [56]. It was assumed that such activity is required to protect snakes from bacteria of their preys [57].

Studies of whole venoms of several snake species revealed their antibacterial activity [58, 59]. For example, the venoms of some African and Asian cobras (genus* Naja*) and some Australian elapids (*Notechis scutatus*,* Pseudechis australis*) display a very prominent antibacterial effect, especially against *Aeromonas hydrophila* [59]. The venoms of one Asian (*N. oxiana*) and one African (*N. melanoleuca*) cobras are an exception and do not display this activity. Gram-negative bacteria of genus* Escherichia coli *show the highest resistance to the effects of all venoms. Gram-negative *Pseudomonas aeruginosa* and Gram-positive *Bacillus subtilis *are less resistant. Gram-positive cocci *Staphylococcus aureus *and Gram-negative bacteria *A. hydrophila *are the most resistant ones. These data show that the venoms affect both Gram-negative and Gram-positive bacteria. In an earlier study [57], it was suggested that *L*-amino acid oxidase, a protein with a molecular weight of ~ 140 kDa present in the venom, is responsible for its antibacterial activity. Later, a number of oxidases from the venoms of different snakes were found to have antibacterial activity (e.g., [60, 61]).

Studies of the antibacterial activity of whole insect and snake venoms against relatively stable *E. coli *bacteria demonstrated increasing efficiency in the series of *Crotalus adamanteus * < *Vipera russellii * < *N. naja sputatrix < Apis mellifera *(honeybee) [62]. According to electron spectroscopy data, the plasma membrane is the main target. Studies of antibacterial activity in a number of snake, scorpion, and bee venoms against Gram-negative bacterium *Burkholderia pseudomallei* showed that the venoms of snakes *C. adamanteus, Daboia russelli russelli, Agkistrodon halys, P. australis, Bungarus candidus, *and *Pseudechis guttatus *display a high activity comparable with that of chloramphenicol and ceftazidime [63]. This remarkably high activity is attributed to the presence of proteins with enzymatic activity, *L*-amino acid oxidase and phospholipase A2, in the venom. It is believed that the oxidative activity of *L*-amino acid oxidase produces hydrogen peroxide that kills bacteria. Introduction of hydrogen peroxide interceptors, such as catalase, abolished the antibacterial activity of the enzyme [64]. Phospholipase A2 cleaves phospholipids, causing membrane permeabilization [65].

The first paper on the antibacterial activity of CT was published in 1968 [66]. It has been reported that a CT extracted from the venom of ringhals *Hemachatus haemachatus *(family *Elapidae*) inhibited *S. aureus *at a concentration of 50 μg/mL. The amino acid sequence of CT was not established at the time. It was known only that the protein has a molecular weight of ~ 7 kD and contains four disulfide bonds.

More detailed information on the antibacterial activity of CTs has been obtained later. In particular, it was found that the CT P4 (amino acid sequence unknown) from *N. nigricollis *is active against several Gram-positive bacteria: *B. subtilus, Micrococcus flavus, Sarcina lutea *[67]. The minimal inhibitory concentrations were in the range of 1.6–6.25 μg/mL. The CT, however, was inactive against Gram-negative bacteria and other microorganisms (yeast, fungi). It can be assumed that CT targets bacterial membranes that contain substantial amounts of anionic phospholipids. Many cytolytic peptides, such as melittin [68] latarcins [69, 70], vaprines [71, 72], and cathelicidin [73], can disrupt the membrane at similar concentrations. Another CT, namely CT3 from *N. atra *(otherwise known as A3, *Table*), has been active not only against Grampositive (*S. aureus*), but also against Gram-negative (*E. coli*) microorganisms [74], even though previous reports indicated no activity for whole *N. atra *venom against *E. coli *[61]. These differences may be explained by the peculiarities of the individual *E. coli* strains that were used in the cited papers. These can only be the differences in the lipopolysaccharide layer of these bacteria (its outer O-antigen portion consisting of branched polysaccharides). Chen *et al*. [74] presented electron microscopy images of bacteria before and after interaction with CT3. It can be seen that the toxin causes characteristic damage to the plasma membrane (protrusions, bubbles and cracks) and, therefore, penetrates through the lipopolysaccharide (LPS) layer. This may occur by substitution of Ca^2+^ ions in the phosphate groups of lipid A via the interaction between the charged side chains of the lysine residues of the toxin and the phosphate groups of lipid A of the LPS, followed by loosening of this layer [75]. An alternative mechanism of cell penetration for antimicrobial peptides through LPS is self-promoted uptake, typical of linear (containing no disulfide bonds) AMPs, such as cecropins [76, 77]. Binding of these peptides to LPS facilitates their penetration into the plasma membrane and increases their membrane-permeabilizing ability. In the case of CT3, some of the molecules remain bound to the LPS, providing other ones with a passage into the plasma membrane. This was demonstrated in experiments with a fluorescent dye leakage from the liposomes formed by phospholipids, whose composition corresponded to that of the phospholipids of the plasma membrane of the bacteria under study [78]. Pre-incubation of CT3 with LPS reduced dye leakage. Thus, the cell wall of Gram-negative bacteria is the major obstacle to the penetration of CTs into the plasma membrane. The high proportion of anionic phospholipids in the plasma membrane enables its destruction by CT molecules. Since the plasma membrane is associated with important cellular functions such as respiration, transportation, osmoregulation, lipid synthesis and others, the loss of its integrity results in cell death [74, 78].

The interaction of CT3 with the cell walls of Grampositive bacteria (mainly with lipoteichoic acid (LTA), which has no polysaccharide moiety) was also examined by Chen *et al*. [74]. Pre-incubation of CT3 with LTA reduced the dye leakage from the liposomes formed from anionic phospholipids (phosphatidylglycerol (PG) : cardiolipin (CL), 6:4) mimicking the plasma membrane of *B. subtilis*. The effective concentration of CT3 (the concentration causing the death of 50% of bacteria) is approximately an order of magnitude smaller (~ 0.9 μM) than that against *E. coli*. This fact most likely indicates that most of the CT3 molecules are not bound to the plasma membranes of these bacteria and are present in the aqueous solution and/or on the outer membrane (LPS), which is a major obstacle for CT molecules. Therefore, CT molecules are too large and conformationally rigid to penetrate this barrier.

As discussed above, the antibacterial action of CTs may be attributed to their membrane activity. To elucidate the mechanism of the destructive action of CT on membranes, Cao *et al*. [79] analyzed the interaction of toxin CT 3 from *N. atra *and toxin gamma from *N. nigricollis* with model membranes of *E. coli *(phosphatidylethanolamine (PE)/PG, 75/25 mol/mol) and *S. aureus* (PG/CL, 60/40 mol/mol). Toxin gamma was equally effective in destroying both PE/PG and PG/CL vesicles. However, CT3 was more effective against PG/CL vesicles. The fusogenic activity of the toxins correlated with their ability to disrupt membranes. For example, CT3, in contrast to toxin gamma, induced a more pronounced membrane fusion with an increase in the cardiolipin content. These data demonstrate that the fusogenic and antibacterial activities of CTs are related.

The attempts to use CT amino acid sequences to design antimicrobial agents that would be smaller but more active than their parent peptides deserve special mention. It was reported earlier that 7- to 12-residue long peptides from loop I of the CT4 of *N. mossambica* display *in vivo *toxicity, albeit lesser than that of the original toxin [80]. The 14-membered cyclic peptide (with one disulfide bond) L1AD3 has the amino acid sequence of loop I of the CT3 of *N. atra *and can induce apoptosis in leukemic T-cells [81, 82] when used at micromolar concentrations. The peptide has a β-hairpin conformation in aqueous solutions, similar to the corresponding moiety within the original CT. Although these short analogs have not been reported to exhibit antibacterial activity, it can be assumed that the β-structural analog possesses this activity. There are several β-structural antimicrobial peptides with one disulfide bond that display a broad range of activities (e.g., [83]). The compact size of these cationic peptides allows them to penetrate through the LPS of Gramnegative bacteria and to destabilize the plasma membrane because of their favorable charge/hydrophobicity ratio. We believe that the emergence of interest in the antibacterial activity of CTs will soon be followed by the development of antimicrobial peptides based on their amino acid sequences.

Notably, the application of computer methods of analysis have allowed researchers to elucidate the evolutionary relationship between animal venom toxins and antimicrobial proteins [84]. It is likely that animal toxins retain their antibacterial function during evolution.

## CONCLUSIONS

The antibacterial activity of CTs varies widely between different members of this family of peptides. The data presented in this review clearly show that penetration through the peptidoglycan layer, bacterial lipopolysaccharide, plays the crucial role in the manifestation of peptide activity. This has been confirmed in a recent study of the comparative activity of five different CTs against some Gram-positive and Gram-negative bacteria [85], which demonstrated that the activity is determined by amino acid residues outside of the CT membrane-binding motif. It might be easier to understand the rules governing the interaction of CTs with the polymers forming the outer membrane and the peptidoglycan layer of a bacterial cell for the peptides whose spatial structure depends on the abundance of disulfide bonds than for mobile linear peptides that have no disulfide bonds. We believe that the next step will be designing peptides based on CTs amino acid sequences. Some steps have already been taken in this direction, and one of the peptides, L1AD3 [81, 82], can be used to treat leukemia. It is likely that there will be more examples in the future.

## Abbreviations

AMP: antimicrobial peptide
GAG: glucosaminoglycan
CL: cardiolipin
LPS: lipopolysaccharide
LTA: lipoteichoic acid
XRA: X-ray analysis
PG: phosphatidylglycerol
PE: phosphatidylethanolamine
CT: cytotoxin (cardiotoxin) from snake venom
NMR: nuclear magnetic resonance
